# The *Wingless* planar cell polarity pathway is essential for optimal activity-dependent synaptic plasticity

**DOI:** 10.3389/fnsyn.2024.1322771

**Published:** 2024-04-03

**Authors:** Carihann Dominicci-Cotto, Mariam Vazquez, Bruno Marie

**Affiliations:** ^1^Department of Anatomy and Neurobiology, Medical Sciences Campus, University of Puerto Rico, San Juan, PR, United States; ^2^Institute of Neurobiology, Medical Sciences Campus, University of Puerto Rico, San Juan, PR, United States; ^3^Molecular Sciences Research Center, University of Puerto Rico, San Juan, PR, United States

**Keywords:** NMJ, disheveled, Rho1, Rac1, Cdc42, Jun kinase, AP1

## Abstract

From fly to man, the Wingless (Wg)/Wnt signaling molecule is essential for both the stability and plasticity of the nervous system. The *Drosophila* neuromuscular junction (NMJ) has proven to be a useful system for deciphering the role of Wg in directing activity-dependent synaptic plasticity (ADSP), which, in the motoneuron, has been shown to be dependent on both the canonical and the noncanonical calcium Wg pathways. Here we show that the noncanonical planar cell polarity (PCP) pathway is an essential component of the Wg signaling system controlling plasticity at the motoneuron synapse. We present evidence that disturbing the PCP pathway leads to a perturbation in ADSP. We first show that a PCP-specific allele of *disheveled* (*dsh*) affects the *de novo* synaptic structures produced during ADSP. We then show that the Rho GTPases downstream of Dsh in the PCP pathway are also involved in regulating the morphological changes that take place after repeated stimulation. Finally, we show that Jun kinase is essential for this phenomenon, whereas we found no indication of the involvement of the transcription factor complex AP1 (Jun/Fos). This work shows the involvement of the neuronal PCP signaling pathway in supporting ADSP. Because we find that AP1 mutants can perform ADSP adequately, we hypothesize that, upon Wg activation, the Rho GTPases and Jun kinase are involved locally at the synapse, in instructing cytoskeletal dynamics responsible for the appearance of the morphological changes occurring during ADSP.

## Introduction

**Figure 1:**
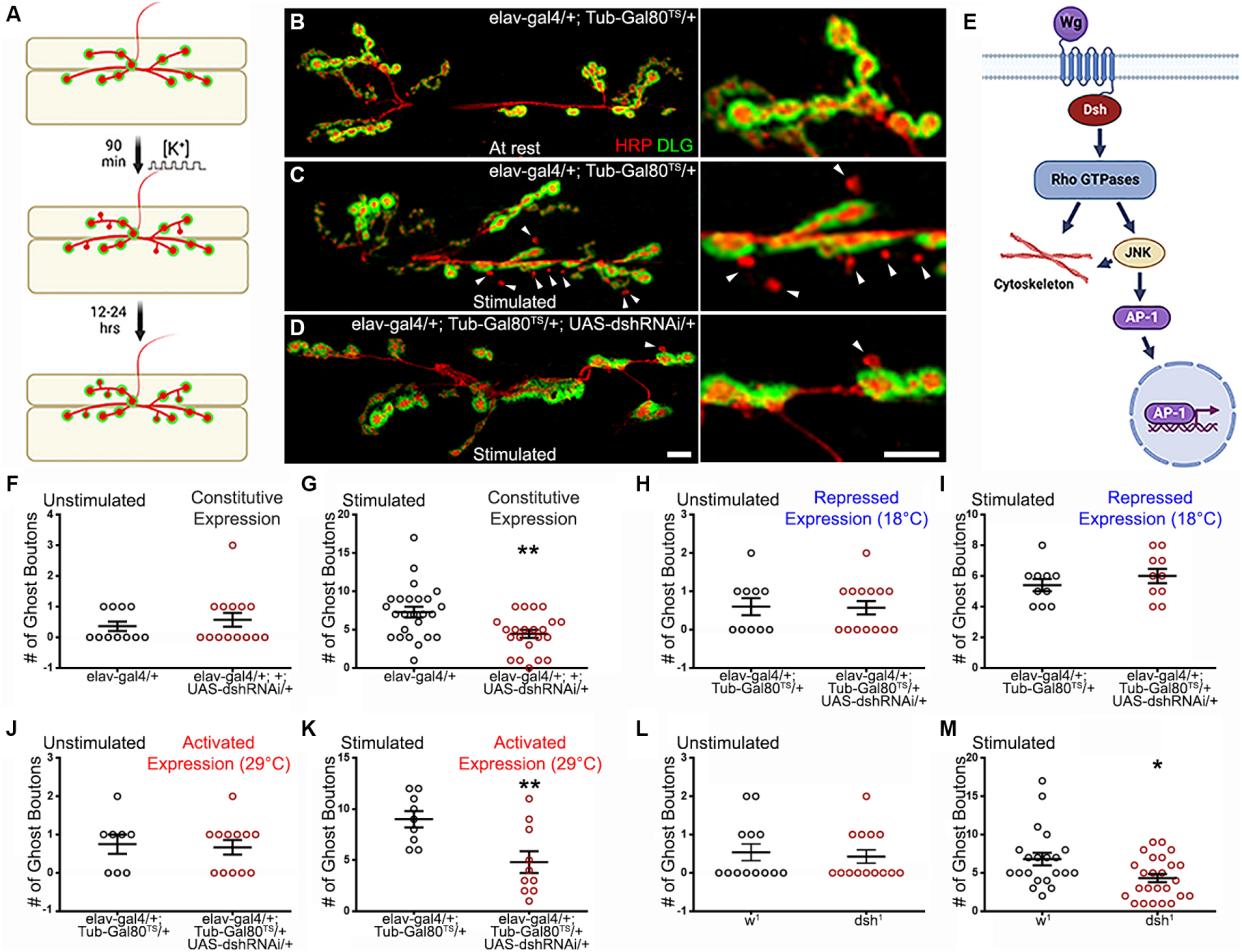
Dishevelled and its planar cell polarity domain regulate ADSP. **(A)** Schematic diagram representing a NMJ labeled with pre-synaptic (red) and post synaptic (green) markers. Upon stimulation, *de novo* synaptic boutons called “ghost boutons” are identifiable because they only show the pre-synaptic marker HRP. These structures, with time, will acquire post-synaptic differentiation. **(B)** Representative confocal photograph of an unstimulated control synapse showing HRP (presynaptic marker) and DLG (postsynaptic marker) immunoreactivity. **(C)** Representative confocal photograph of a stimulated control synapse. Arrowheads point to ghost boutons. **(D)** Representative confocal photograph of a stimulated synapse expressing *dsh* RNAi. **(E)** Schematic diagram illustrating the components of the Wg planar cell polarity pathway. **(F–M)** Quantification of the ghost bouton number in **(F)** unstimulated and **(G)** stimulated control and preparations expressing *dsh* RNAi constitutively; **(H)** unstimulated and **(I)** stimulated control and preparation carrying the suppressed conditional expression system at 18°C; **(J)** unstimulated and **(K)** stimulated control and preparation expressing *dsh* RNAi for 48 h prior to the stimulation (activated expression system at 29°C); **(L)** unstimulated and **(M)** stimulated control and dsh^1^ mutant preparations. Scale bar: 10 μm. **(F,H)** Mann–Whitney test. **(G,I–M)** Two-tailed, unpaired *t*-tests: *p < 0.05; **p < 0.01. Data are shown as scatter plots and means ± SEM.

Synapses are the site of plastic events that shape their morphological and electrophysiological properties depending on their experiences (Shepherd, 2004; Holtmaat and Svoboda, 2009; Ho et al., 2011; Pereda, 2014; Harris and Littleton, 2015). One such plastic event is activity-dependent synaptic plasticity (ADSP), by which the synaptic efficacy can be increased or decreased, reflecting previous excitatory or inhibitory stimuli (Castillo, 2012; Lüscher and Malenka, 2012). This plasticity is thought to be the cellular correlate of learning and memory, and it is essential to improve our understanding of the molecules and molecular signals underlying this phenomenon. The secreted molecule Wg/Wnt regulates ADSP (Budnik and Salinas, 2011; Rosso et al., 2013). Indeed, activity-dependent mechanisms induce Wnt release that, in turn, induces plasticity signaling in hippocampal neurons (Chen et al., 2006; Tabatadze et al., 2014). This increase in Wnt secretion provokes structural alterations in dendritic arborizations (Yu and Malenka, 2003; Wayman et al., 2006; Ferrari et al., 2018) and spines (Ciani et al., 2011; Tabatadze et al., 2014; McLeod et al., 2018), promoting changes in synaptic strength and plasticity. *Drosophila* served as a model of choice to explore the instrumental role of Wg in directing ADSP (Budnik and Salinas, 2011; Bai and Suzuki, 2020). ADSP can be elicited at the NMJ when the preparation is submitted to a repeated stimulation protocol (Ataman et al., 2008; Piccioli and Littleton, 2014; Vasin et al., 2014, 2019; Maldonado-Díaz et al., 2021), similar to the one used on hippocampal neurons to elicit LTP (Wu et al., 2001). The changes associated with the NMJ experiencing ADSP are both electrophysiological and structural (Ataman et al., 2008; Alicea et al., 2017; Maldonado-Díaz et al., 2021). Live and confocal imaging, electron microscopy and tomography at the NMJ allowed for the observation of these structural changes (Ataman et al., 2008; Vasin et al., 2019). Two types of synaptic modifications appeared very quickly (within 1 h) after repeated stimulation: filipodia-like structures called synaptopods, and newly formed varicosities containing synaptic vesicles but devoid of postsynaptic structures, called ghost boutons (Figure 1A). These ghost boutons develop within 24 h into mature boutons by acquiring presynaptic active zone markers and postsynaptic glutamate receptors. Because they are easily identifiable, ghost boutons are used to assess ADSP. The Wg canonical pathway is essential for this plasticity (Ataman et al., 2008). Repeated stimulation of the NMJ promotes an increase in synaptic Wg that leads to rapid structural and electrophysiological changes (Ataman et al., 2008). In fact, Wg signaling regulates these changes bidirectionally; post-synaptically through nuclear import of its receptor, and pre-synaptically by the inhibition of GSK3ß/Sgg (Ataman et al., 2008). In addition, there is evidence that the calcium pathway is involved in this plasticity. In this pathway, an increase in intracellular calcium activates CaMKII and the transcription factor NFAT (Koles and Budnik, 2012). The neuronal expression of NFAT subunit A at the NMJ prevents the formation of ghost boutons upon stimulation (Freeman et al., 2011). In addition, synaptic CaMKII protein expression increases upon stimulation which correlates with *de novo* bouton formation (Nesler et al., 2016). However, to date the third pathway that responds to the Wg/Wnt signal, the planar cell polarity (PCP) pathway, has not yet been assessed for its role in regulating ADSP.

Here we use the *Drosophila* NMJ model to ask whether the Wg PCP pathway (Figure 1E) is involved in ADSP. We use the appearance of *de novo* synaptic structures, ghost boutons, to quantify the extent of these changes. Because of their rapid development (90 min following stimulation), ghost boutons represent early pre-synaptic structures that are devoid of post-synaptic differentiation (Figure 1A). This characteristic has been widely used to easily identify ghost boutons at the NMJ, in order to quantify ADSP (Ataman et al., 2008; Fuentes-Medel et al., 2009; Freeman et al., 2011; Nesler et al., 2013, 2016; Piccioli and Littleton, 2014; Vasin et al., 2014, 2019; Alicea et al., 2017; Maldonado-Díaz et al., 2021). Our present work investigates the role of the PCP pathway in the production of these morphological synaptic changes. We find that several pathway members are involved in this process. Indeed, we first show that a PCP-specific *dsh* allele (Perrimon and Mahowald, 1987) is necessary for optimal ADSP. We then show that activity of the Rho1, Rac1, and Cdc42 GTPases can also influence ADSP. We then investigate the Jun kinase (JNK), an effector of these GTPases (Coso et al., 1995; Rosso et al., 2005) and show that its loss of function is detrimental to ADSP; however, the transcription factor AP1 (Fos/Jun), a target of JNK (Sanyal et al., 2002; Etter et al., 2005), is not necessary for this process. In most of our experiments, we use an inducible RNA expression system to selectively down- or up-regulate the activity of the members of the PCP pathway pre-synaptically only (post-synaptic expression is unchanged) and late in development (after the establishment of the NMJ). This allows us to conclude that the effects we observed are due to a change in the PCP pathway activity within the motoneuron and not a consequence of developmental perturbation. On the contrary, we argue that it reflects a need for the PCP pathway within the motoneuron to achieve optimal ADSP.

## Materials and methods

### Genetics

For experiments using the *elav^c155^-gal4* insertion on the X chromosome, only females were used (in order to consider heterozygote animals). In the other experimental procedures, animals from both sexes were used. Flies were reared on standard fly food at 18°C for conditional experiments or at 25°C in all other cases. Our methods are consistent with standard husbandry and care. Our laboratory follows ethical practices while carrying out experiments as well as the cataloging of *Drosophila*. The fly strains used in this study include *dsh^1^* [Bloomington Drosophila stock center (BDSC), stock #5298], and *w^1^* (BDSC, stock #145) as a control. We used the Gal4/UAS system (Brand and Perrimon, 1993) to express dominant-negative or constitutively active genes in neurons. For this, we used the pan-neuronal driver *elav^c155^-gal4* (BDSC, stock #458) whose expression starts early in embryogenesis (after neuroblast mitosis) in combination with *UAS-dsh-RNAi* (BDSC, stock #31306), *UAS-rac1^N17^* (BDSC, stock #6292), *UAS-rac1^V12^* (BDSC, stock #6291), *UAS-rho1^N19^* (BDSC, stock #58818), *UAS-rho1^V12^* (BDSC, stock #58817), *UAS-cdc42^N17^*(BDSC, stock #6288), *UAS-cdc42^V12^* (BDSC, stock #4854), *Tub-Gal80^TS^* (BDSC, stock #7108), *UAS-jnk^DN^* (BDSC, stock #9311), *UAS-fos^DN^* (BDSC, stock #7215) and *UAS-jun^DN^* (BDSC, stock #7218). The controls for these experiments were the driver elav^c155^-gal4/+. For the conditional experiments, we made a fly containing the *elav^c155^-gal4* on the first chromosome and *Tub-Gal80^TS^* on the second chromosome. This fly expresses the thermosensitive inhibitor Gal80^TS^ (McGuire et al., 2004) under the control of an ubiquitous *tubulin* (*Tub*) promoter. Larvae for the conditional experiments were reared at 18°C, where Gal80^TS^ inhibits protein expression by binding to the Gal4 activator. Then, we shifted them to 29°C, inhibiting Gal80^TS^ and therefore allowing the expression of specific proteins activated by Gal4. Considering the lethality and the axon pathfinding errors associated with the early expression of these transgenes, we decided to limit their expression to 48 h before performing the stimulation protocol. At this time, we minimize the effect of the transgenes’ expression on synapse development and growth while affecting their function for ADSP.

### Stimulation protocol

We dissected the body wall muscles from third instar larvae in hemolymph-like HL3 saline (70 mM NaCl, 10 mM NaHCO3, 115 mM sucrose, 5 mM trehalose, 5 mM HEPES, 10 mM MgCl2), leaving unharmed the CNS and the peripheral nerves innervating the body wall muscles. The protocol was carried out on partially intact and non-stretched preparations. The stimulation protocol that we used was adapted from Ataman et al. (2008) and is constituted of 5 stages that alternate stimulation and rest periods. In the first three stages the preparations were stimulated for 2 min followed by a 15 min rest period. The fourth stage is composed of a 4 min stimulation period followed by a 15 min rest while the fifth and last stage is composed of a 6 min stimulation period followed by a 14 min rest. The 5 stimulation periods were elicited using a potassium- and calcium-rich HL3 saline solution (90 mM KCl and 1.5 mM CaCl2), while during the five rest periods a HL3 saline containing 5 mM KCl and 0.1 mM CaCl_2_ was applied.

### Immunohistochemistry

After stimulation, the preparations were fixed for 15 min at room temperature in a solution of 4% paraformaldehyde in PBS before being incubated overnight at 4°C within a primary (anti-Dlg) antibody solution (1:20; Budnik et al., 1996). The secondary antibody (1,300; Alexa Fluor 488-conjugated AffiniPure goat anti-mouse or anti-rabbit, Jackson ImmunoResearch) and Anti-Hrp (1,300; Cy3-conjugated AffiniPure goat anti-horseradish peroxidase, Jackson ImmunoResearch; Jan and Jan, 1982) were applied for 1 h at room temperature as previously described (Alicea et al., 2017; Maldonado-Díaz et al., 2021).

### Quantification of ghost boutons

We treated control preparations in parallel with each experimental genotype to account for the potential variation in our experimental manipulations. The control preparations were designed to contain the experimental preparations’ genetic background (*w^1^* for *dsh^1^* or the corresponding *gal4* driver for the different expression systems used). Control and experimental preparations were also reared at the same temperature and submitted to the same shift (18–29°C) when applicable. We identified a ghost bouton as a bouton that had a positive immunoreactivity to anti-HRP and negative immunoreactivity to anti-Dlg. In all the experiments, ghost boutons were counted on muscle 6/7 NMJs, in A3 segments of third instar larvae. To carry out these observations, we used a Nikon Eclipse 80i microscope at a magnification of 400X.

### Statistical treatment

We first identified any outlier using the ROUT method with a Q = 0.1%. Then, we assessed whether the data fitted a normal distribution using the Shapiro–Wilk normality test. For data sets with *n* ≥ 12, parametric tests were run when the Shapiro–Wilk normality test showed *p* > 0.0001. For data sets with *n* < 12, parametric tests were run when the Shapiro–Wilk normality test showed *p* > 0.05. For cases of parametric statistics, we ran an ANOVA test—when there are three or more data sets. We applied a *post hoc* Dunnett’s correction test when multiple comparisons are carried out against a control value. When only two data sets were compared, we performed two-tailed, unpaired t-tests. For non-parametric tests and when comparing three or more data sets, we ran a Kruskal–Wallis test with a *post hoc* Dunn’s multiple comparison test. When comparing two data sets, the Mann-Witney test was used. GraphPad Prism 6 was used to apply these statistical treatments.

## Results

### Disheveled and its PCP-specific domain relay the signal necessary for ADSP

Previous studies showed that the secreted molecule Wg and its pre-synaptic receptor Fz2 controlled ADSP at the *Drosophila* NMJ (Ataman et al., 2008; Alicea et al., 2017). Because Disheveled (Dsh) is the cytoplasmic molecule making the link between the Wg receptor and its different effectors, it is often called the Wg hub (Gao and Chen, 2010). We asked whether Dsh loss of function could reduce ADSP and phenocopy the defects in ADSP observed in the *wg* and *fz2* loss of function backgrounds (Ataman et al., 2008; Alicea et al., 2017). We used a well-described method to repeatedly stimulate the NMJ preparation in a way reminiscent of the stimulation protocol used on hippocampal neurons in culture (Wu et al., 2001). In these conditions, after a 90-min patterned repeated stimulation protocol, we observe the appearance of ghost boutons (see schematic diagram Figure 1A; Ataman et al., 2008) that show the neuronal membrane-specific marker revealed by the anti-HRP immunoreactivity (Jan and Jan, 1982), without the apposition of the post-synaptic marker Dlg (Disc large, the membrane-associated guanylate kinase homolog; Lahey et al., 1994; Budnik et al., 1996). These *de-novo* synaptic structures are noticeable when comparing unstimulated control synapses (Figure 1B) to stimulated control synapses (Figure 1C; arrowheads point to ghost boutons) and are easily quantifiable (Figures 1F–M).

Throughout this manuscript, we paid attention to the status of the synapse at rest (without stimulation) to ensure that there was no significant difference in the mean synaptic ghost bouton number between the studied genetic backgrounds. This way, we can infer that any observed differences after stimulation reflect a change in the synaptic answer to the activity-dependent process. In fact, unstimulated preparations across all the genotypes studied (Figures 1F,H,J,L) show no differences. We later ensured that our stimulation protocol was efficient in eliciting ghost boutons. Here, our unstimulated control preparations showed a mean of 0.36 ± 0.15 ghost boutons per synapse (Figure 1F, black circle, *n* = 11), and our stimulated controls showed 7.28 ± 0.69 ghost boutons (Figure 1G, black circle, *n* = 25), indicating an increase in ghost boutons comparable to previously published work (Ataman et al., 2008; Freeman et al., 2011; Nesler et al., 2013, 2016; Piccioli and Littleton, 2014; Vasin et al., 2014, 2019; Alicea et al., 2017; Lee et al., 2017; Maldonado-Díaz et al., 2021).

We then asked whether Dsh was necessary to perform ADSP. Because of the importance of Dsh in transducing the Wg signal, its null alleles (for example *dsh^X788^* and *dsh^nYn234Y^*) are embryonic lethal and therefore do not allow the study of the larval NMJ (Perrimon and Mahowald, 1987; Klingensmith et al., 1994). To create a viable loss of function condition for *dsh*, we used RNA interference (RNAi; Piccin et al., 2001) and expressed it using the *elav-Gal4* post-mitotic neuronal driver (Robinow and White, 1991). When these preparations were submitted to the patterned repeated stimulation protocol, they showed fewer ghost boutons (Figure 1G, red circles). The mean ghost bouton number in these preparations was 4.46 ± 2.48, a value less than the control value (*p* = 0.0026; *n* = 22), suggesting that Dsh is indeed involved in ADSP.

Although we used the postmitotic driver *elav-Gal4* to initiate *dsh* loss of function, its expression starts early in development (end of embryogenesis). Therefore, the phenotype we observe could be caused by a detrimental event during synapse formation due to a lack of Dsh activity. To rule out this possibility, we first examined the NMJ and did not notice any phenotype affecting axon routing or synaptic growth. Secondly, we designed an inducible expression system to affect Dsh activity. This system involves the expression of 3 different transgenes within the studied animal: the *elav-Gal4* driver, the effector *UAS-dsh-RNAi*, and the inhibitor *Tub-Gal80^TS^*. In this system, the temperature-sensitive (TS) Gal80^TS^ protein is expressed ubiquitously (under the control of the tubulin promoter) and able to repress Gal4 at permissive temperature (18°C; McGuire et al., 2004). Indeed, at this temperature, the expression of *dsh* RNAi is shut off and we observe no difference between the controls and the animals containing *dsh* RNAi in both non-stimulated (Figure 1H) and stimulated (Figure 1I) preparations. At the restrictive temperature (29°C), the temperature-sensitive mutation carried by Gal80^TS^ is revealed, producing a non-functional Gal80, which leads to the de-repression of the system, and the expression of *dsh* RNAi. In this condition, we can observe a loss of function phenotype when we elicit ADSP. When we derepressed the Gal4/UAS system for 48 h before performing the stimulus protocol, we did not observe any difference at rest between controls and dsh RNAi loss of function (the mean value of controls is 0.75 ± 0.25, *n* = 8, while it is 0.67 ± 0.19, *n* = 12 for dsh RNAi expressing animals; Figures 1B,J). After repeated patterned stimulations, we did observe differences between controls and preparations containing the *dsh* RNAi construct. While the mean value of ghost boutons is 9 ± 0.8 for controls (*n* = 9; Figures 1C,K), it is reduced in dsh RNAi animals (4.8 ± 1.07 ghost boutons; *n* = 10; *p* = 0.007; Figures 1D,K). This reduction, provoked by a loss of Dsh activity only 48 h before applying the repeated stimulation protocol, is comparable to the reduction observed when we inhibit Dsh function since the end of embryogenesis (Figure 1G). This suggests that the effect observed on ADSP is due to the requirement of Dsh function at the time of the stimulation challenge and not a consequence of perturbed development. It is important to note that we have previously shown that temperature itself can have limited yet significant effects on the apparition of ghost boutons (Maldonado-Díaz et al., 2021). This is why our work here will only compare the data emanating from animals that experienced identical temperatures during their development and experimental procedures.

Having established the importance of Dsh in transducing the signal responsible for ADSP, we asked whether the planar cell polarity pathway (PCP) was involved in this phenomenon. To do so, we took advantage of the previously studied and isolated *dsh^1^* allele (Perrimon and Mahowald, 1987). This allele, often referred to as a PCP-specific allele, encodes a version of Dsh that selectively affects the transduction of the Wg signaling to the PCP pathway without altering the efficacy of the Wg signal toward the canonical or the calcium pathway (Axelrod et al., 1998; Boutros et al., 1998; Yanfeng et al., 2011). At rest, there is no difference between control and *dsh^1^* animals (the mean value of controls and *dsh^1^* animals are 0.54 ± 0.21, n = 13 and 0.55 ± 0.8, *n* = 14 respectively; Figure 1L). Nevertheless, after repeated patterned stimulation, the mean number of ghost boutons appearing at the synapse is reduced in *dsh^1^* animals (4.32 ± 0.54, *n* = 25; red circles, Figure 1M) when compared to control (6.81 ± 0.83, *n* = 21; *p* = 0.017; black circles, Figure 1M). This result suggests that part of the Wg signal required to perform ADSP is transduced through the PCP pathway. To consolidate the idea that the Wg PCP pathway is an integral part of ADSP machinery, we decided to ask whether other members of this pathway (Figure 1E) could affect this plasticity.

### Rho1 activity can repress the morphological changes associated with ADSP

Since Dsh transduces the Wg signal through several GTPases (Boutros et al., 1998; Fanto et al., 2000; Schlessinger et al., 2007), we decided to investigate the role of Rho1 in ADSP. Rho1 is a small GTPase that functions as a molecular switch cycling between an inactive GDP-bound form and an active GTP-bound form to mainly regulate the assembly and reorganization of the actin cytoskeleton controlling different cellular activities (Jaffe and Hall, 2005). In this way, Rho1 influences several biological processes such as embryogenesis, cell polarity, and cell division (Hariharan et al., 1995; Olson et al., 1995; Barrett et al., 1997; Strutt et al., 1997). Its role in neuronal development has also been studied; Rho activation can promote or inhibit neuronal growth cones as well as inhibit dendritic growth (Luo, 2000; Dickson, 2001; Auer et al., 2011). Reducing the activity of p190 RhoGAP, a negative regulator of Rho, provokes axonal guidance defects and decreases axon number in hippocampal cells (Brouns et al., 2001). In Drosophila mushroom bodies, the reduction of p190 RhoGAP promotes retraction of axonal branching, while its overexpression promotes axonal extension (Billuart et al., 2001). In addition, inhibition of neural activity in cortical neurons decreases active Rho levels while expression of constitutively active Rho increases axon branching by elimination and addition of branches (Ohnami et al., 2008).

**Figure 2:**
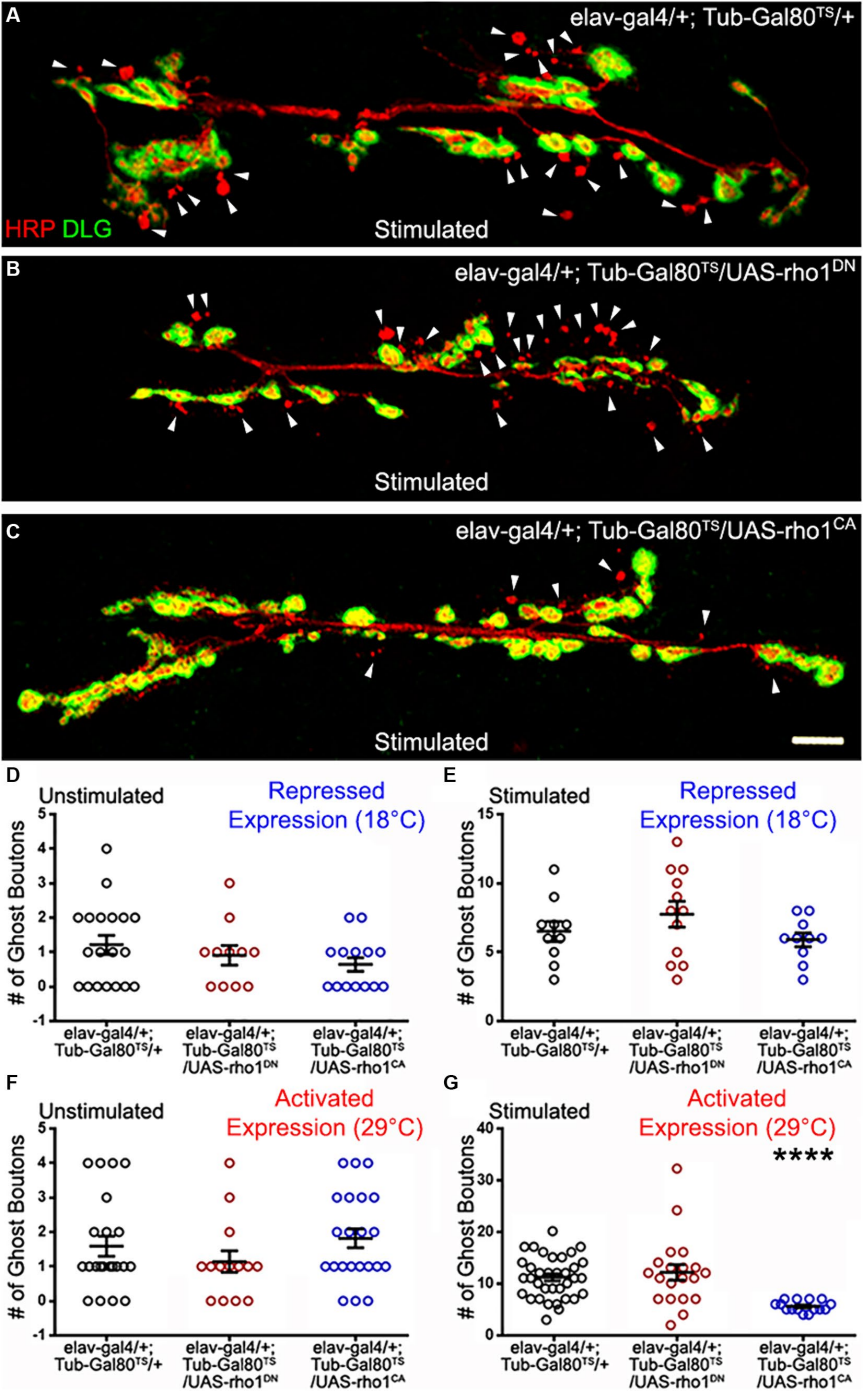
The GTPase Rho1 can repress ADSP. **(A)** Representative confocal photograph of a stimulated control synapse showing HRP and DLG immunoreactivity and ghost bouton formation (arrowheads). **(B)** Representative confocal photograph of a stimulated synapse expressing a dominant negative (DN) form of the Rho1 GTPase. **(C)** Representative confocal photograph of a stimulated synapse expressing a constitutively active (CA) form of the Rho1 GTPase. **(D–G)** Quantification of mean ghost bouton number in **(D)** unstimulated and **(E)** stimulated preparations containing the repressed (18°C) conditional expression system to express Rho1^DN^ or Rho1^CA^; **(F)** unstimulated and **(G)** stimulated preparations expressing (29°C) Rho1^DN^ or Rho1^CA^ 48 h prior to the stimulation. Scale bar: 10 μm. **(D,G)** Kruskal–Wallis with Dunn’s multiple comparison test. **(E,F)** ANOVA with Dunnett’s multiple comparisons test: ****p < 0.0001. Data are shown as scatter plots and means ± SEM.

Here we asked whether Rho1 was involved in ADSP at the NMJ. Because of Rho1’s multiple cellular functions its null mutations are lethal, so we therefore decided to express a dominant negative version (Rho1^DN^, see material and methods) and a constitutively active version (Rho1^CA^, see material and methods). We used the Gal4/Gal80^TS^ inducible system described previously to bypass the potential effects of these constructs’ expression on axon pathfinding and neuronal growth. We first checked that there were no notable differences regarding the number of ghost boutons in unstimulated preparations across all the genotypes considered at both permissive temperature (controls showed 1.21 ± 0.27 ghost boutons; *n* = 19; Rho1^DN^ 0.91 ± 0.28; *n* = 11; *p* = 0.57; Rho1^CA^ 0.64 ± 0.2; *n* = 14; *p* = 0.2; Figure 2D) and restrictive temperature (control showed 1.6 ± 0.29 ghost boutons; *n* = 22; Rho1^DN^ 1.14 ± 0.31; *n* = 14; *p* = 0.6; Rho1^CA^ 1.82 ± 0.28; *n* = 22; *p* = 0.97; Figure 2F). We also asked whether the inhibition of the Rho1^DN^ or Rho1^CA^ expression by Gal80^TS^ at 18°C was consistent with the observed results; we expected all the preparations to behave like controls if the expression was silenced efficiently. Indeed, the stimulated control preparations showed a mean number of ghost boutons of 6.5 ± 0.73 (Figure 2E; *n* = 10), a number not statistically different from the one observed in the stimulated preparations carrying the silenced dominant negative construct (7.75 ± 0.93; *n* = 12; *p* = 0.42) or the silenced constitutively active construct (5.9 ± 0.5; *n* = 10; *p* = 0.82). This suggests that the expression of these transgenes is efficiently suppressed by Gal80^TS^ at 18°C or that it does not affect the mean ghost bouton number. Notably, we can confirm that our constructs worked effectively because the expression of either Rho1^DN^ or Rho1^CA^ at 25°C was lethal. We therefore shifted the temperature to 29°C 48 h before assessing ADSP. In this condition, we observed a mean number of ghost boutons of 11.14 ± 0.68 (Figure 2A,G; *n* = 35) in control animals, while animals expressing the dominant negative form of Rho1 presented a mean ghost bouton number of 12.10 ± 1.5 (*n* = 20), a value similar to control (Figure 2B,G; *p* > 0.99). This suggests that Rho1 activity is not required for the formation of the morphological synaptic structures associated with ADSP. In contrast, when we expressed the constitutively active version of Rho1 in neurons 48 h before repeated stimulation we could observe a decrease in the mean number of GB formed (Figure 2C,G; 5.6 ± 0.27; *n* = 15; *p* < 0.0001). These results show that the Rho1 GTPase activity can suppress part of the morphological synaptic response that is associated with activity-dependent synaptic plasticity. This is reminiscent of its role in neuronal morphogenesis and structural plasticity in which Rho1 signaling regulates repulsive axon guidance cues and axon or dendritic retraction (Luo, 2002).

### The GTPase Cdc42 represses ADSP

**Figure 3:**
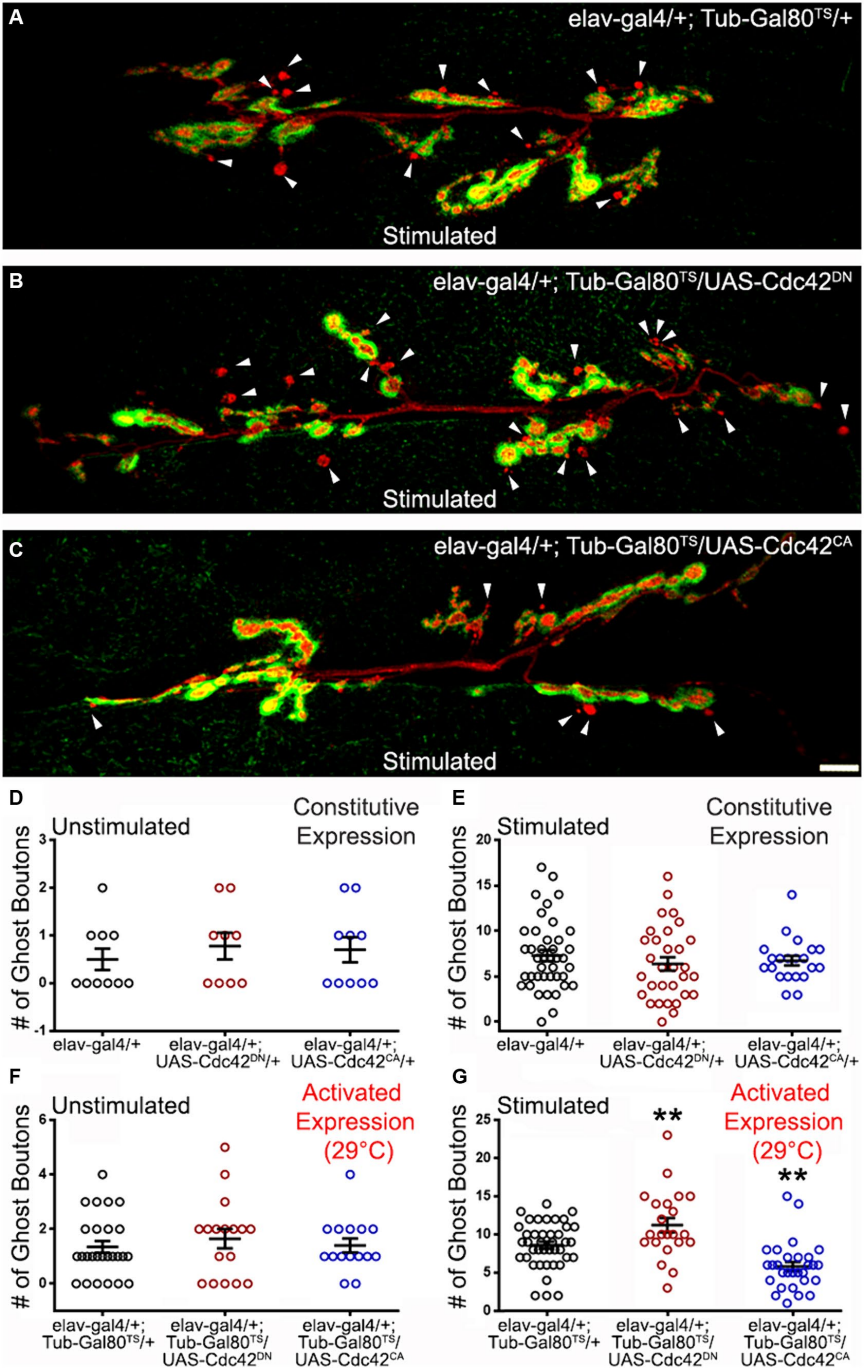
The GTPase Cdc42 represses ADSP. **(A)** Representative confocal photograph of a stimulated control synapse showing HRP and DLG immunoreactivity and ghost bouton formation (arrowheads). **(B)** Representative confocal photograph of a stimulated synapse expressing a dominant negative (DN) form of the Cdc42 GTPase. **(C)** Representative confocal photograph of a stimulated synapse expressing a constitutively active (CA) form of the Cdc42 GTPase. **(D–G)** Quantification of mean ghost bouton number in **(D)** unstimulated and **(E)** stimulated control and preparations expressing Cdc42^DN^ or Cdc42^CA^ constitutively; **(F)** unstimulated and **(G)** stimulated control and preparations expressing Cdc42^DN^ or Cdc42^CA^ for 48 h prior to the stimulation. Scale bar: 10 μm. **(D)** Kruskal–Wallis with Dunn’s multiple comparison test. **(E–G)** ANOVA with Dunnett’s multiple comparisons test: **p < 0.01. Data are shown as scatter plots and mean ± SEM.

We then investigated the role of another GTPase, Cdc42, known to be activated downstream of Dsh (Moriguchi et al., 1999; Schlessinger et al., 2007). We first constitutively expressed dominant negative (DN) or constitutively active (CA) versions of Cdc42 in post-mitotic neurons (*elav-Gal4* driver). Under these conditions, we did not observe any differences in the mean number of ghost boutons in unstimulated and stimulated preparations (Figures 3D,E). This suggests that changing Cdc42 activity might have no consequences on the neuronal properties underlying ADSP or that these consequences might be compensated for during development. To distinguish between these two possibilities, we used the previously described inducible expression system in which expression of the altered Cdc42 constructs is switched on 48 h before carrying out the repeated stimulation protocol. As previously described, we confirmed again that the system was inhibited at 18°C (data not shown) before de-repressing the expression system 48 h prior to the repeated stimulation protocol. Following this activation, we observed no change in unstimulated preparations (Figure 3F). In contrast, after repeated stimulations we observed an increase in the mean number of ghost boutons when we expressed the dominant negative form of Cdc42 (compare 8.61 ± 0.46 in control to 11.23 ± 4.46 in cdc42^DN^; *p* = 0.009; Figures 3A,B,G). Interestingly, the expression of the constitutively active form of Cdc42 showed a decrease in the number of morphological changes associated with ADSP (average of ghost bouton is 5.83 ± 0.58 in cdc42CA; *p* = 0.002; Figures 3C,G). These results suggest that Cdc42 is involved in repressing the expression of ADSP.

### The GTPase Rac 1 affects ADSP

**Figure 4:**
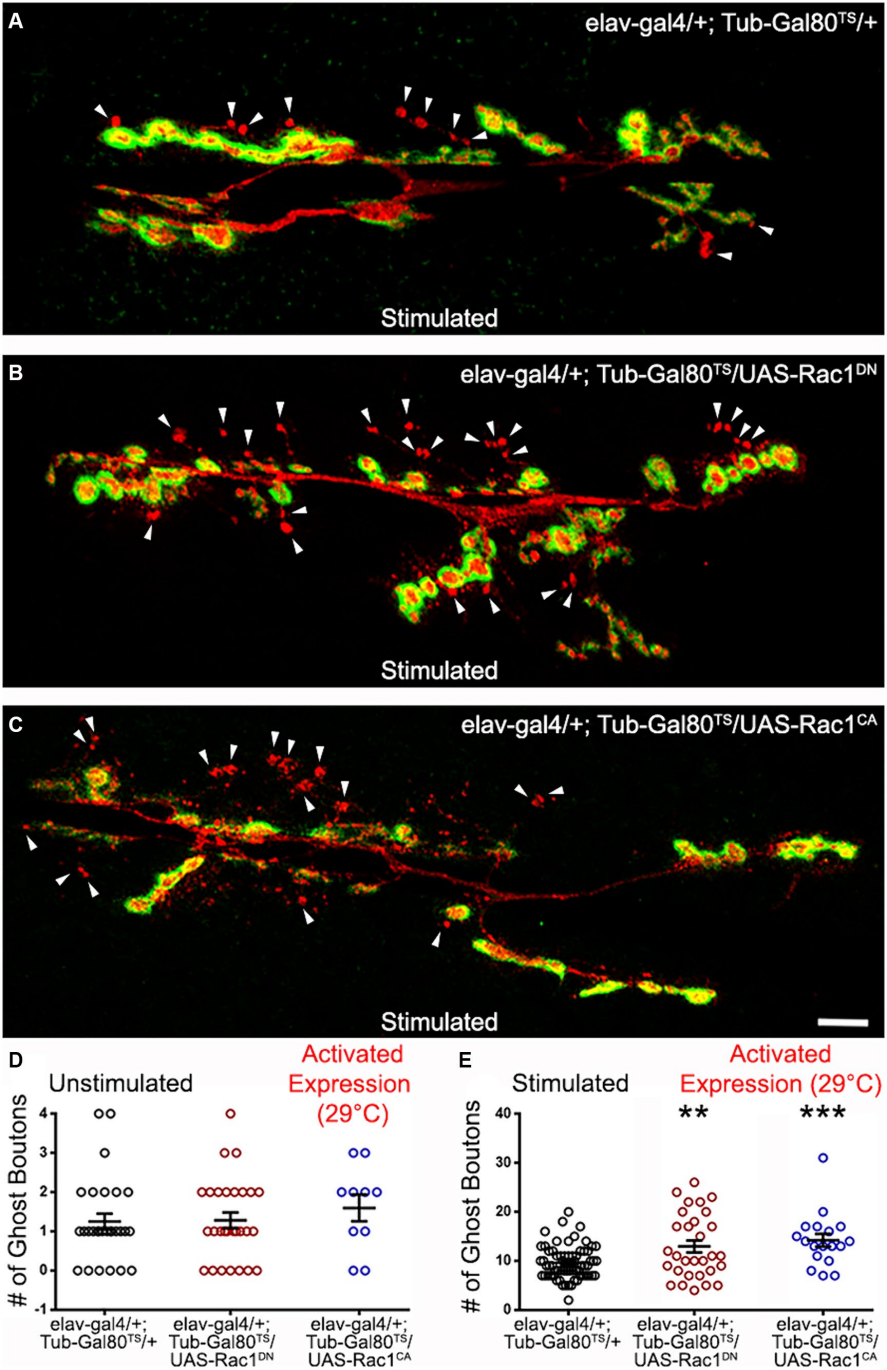
The GTPase Rac 1 can regulate ADSP. **(A)** Representative confocal photograph of a stimulated control synapse showing HRP and DLG immunoreactivity and ghost bouton formation (arrowheads). **(B)** Representative confocal photograph of a stimulated synapse expressing a dominant negative (DN) form of the Rac1 GTPase. **(C)** Representative confocal photograph of a stimulated synapse expressing a constitutively active (CA) form of the Rac1 GTPase. Quantification of mean ghost bouton number in **(D)** unstimulated and **(E)** stimulated control and preparations expressing Cdc42^DN^ or Cdc42^CA^ for 48 h prior to the stimulation. Scale bar: 10 μm. ANOVA with Dunnett’s multiple comparisons test: **p < 0.01; ***p < 0.001. Data shown as scatter plots and mean ± SEM.

We tested a third GTPase activated by Dsh and the Wg signaling, Rac1 (Fanto et al., 2000; Bikkavilli et al., 2008; Li et al., 2016), for its potential role in shaping activity-dependent synaptic plasticity. As previously described, we used a temperature-driven conditional expression system to express Rac1 dominant negative (DN) or constitutively active (CA) versions. We first verified that no phenotypes were associated with Rac1 expression at low temperatures (18°C; data not shown). We then induced Rac1^DN^ or Rac1^CA^ expression 48 h before our experimental procedures. While we observed no differences in unstimulated preparations (Figure 4D), we did observe changes in the mean number of ghost boutons present at the Rac1^DN^- and Rac1^CA^- expressing synapses. When control synapses showed a mean number of ghost boutons of 9.62 ± 0.42 (dark circles; *n* = 65; Figure 4A), synapses expressing both Rac1 constructs showed an increase in the mean ghost bouton number. Indeed, Rac1^DN^ expressing synapses showed 12.93 ± 1.2 ghost boutons (red circles; *n* = 30; *p* = 0.002; Figures 4B,E) and Rac1^CA^ expressing synapses showed 14.2 ± 1.3 (blue circles; *n* = 18; *p* = 0.0009; Figures 4C,E). Although it seems counterintuitive that the expression of both the dominant negative and the constitutively active forms of Rac1 provokes the same phenotype it is reminiscent of phenotypes previously observed during axon pathfinding within neurons expressing Rac1^DN^ or Rac1^CA^. Indeed, ISNb axons of motoneurons expressing either Rac1^DN^ or Rac1^CA^ during embryonic development showed arrested growth cones (Kaufmann et al., 1998). In addition, the expression of both Rac1^DN^ and Rac1^CA^ during embryonic axonal outgrowth causes increased axonal loss (Luo et al., 1994). Interestingly, that study showed that Rac1^CA^ expression induced a stronger phenotype and an accumulation of F-actin that was not present in Rac1^DN^-expressing animals (Luo et al., 1994), suggesting that Rac1^CA^ and Rac1^DN^ expression led to the same phenotype by affecting different cytoskeletal processes. Interestingly, at the NMJ expression of Rac1^DN^ (Park et al., 2022) and Rac1^CA^ (Ball et al., 2010) both cause an increase of synaptic boutons. Here we show that changing Rac1 activity increases the morphological changes associated with activity-dependent synaptic plasticity. Taken together, these results show that the Rho family GTPases downstream of Dsh are involved in the morphological response associated with activity-dependent synaptic plasticity.

### Jun kinase activity is required for optimal ADSP in an AP1-independent manner

**Figure 5:**
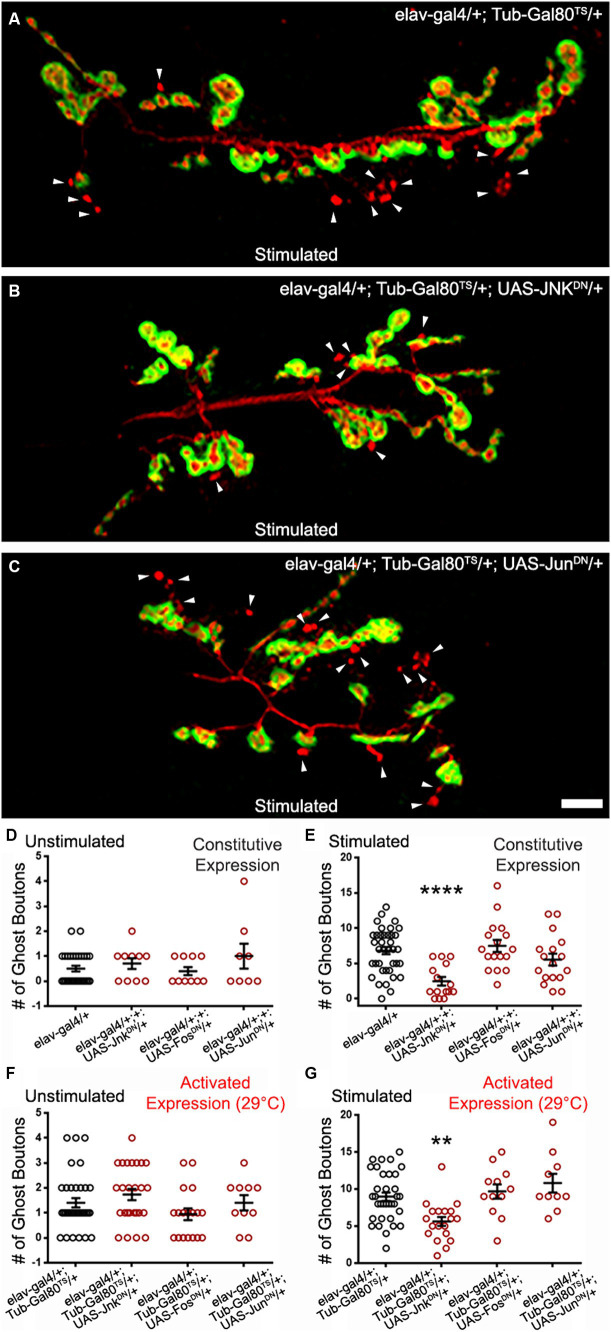
Jun Kinase is required for optimal ADSP in an AP1-independent manner. **(A)** Representative confocal photograph of a stimulated control synapse showing HRP and DLG immunoreactivity and ghost bouton formation (arrowheads). **(B)** Representative confocal photograph of a stimulated synapse expressing a dominant negative (DN) form of Jun Kinase. **(C)** Representative confocal photograph of a stimulated synapse expressing a dominant negative (DN) form of c Jun. **(D–G)** Quantification of mean ghost bouton number in **(D)** unstimulated and **(E)** stimulated control and preparations constitutively expressing Jnk^DN^ or c Jun^DN^ or c Fos^DN^; **(F)** unstimulated and **(G)** preparations expressing Jnk^DN^ or c Jun^DN^ or c Fos^DN^ for 48 h prior to the stimulation. Scale bar: 10 μm. **(D,F,G)** Kruskal–Wallis with Dunn’s multiple comparisons test **(E)** ANOVA with Dunnett’s multiple comparisons test: **p < 0.01; ****p < 0.0001. Data are shown as scatter plots and mean ± SEM.

One of the major PCP pathway effectors is Jun kinase (Jnk; Strutt et al., 1997; Boutros et al., 1998; Jenny, 2010). This kinase can be activated by the Rho GTPases and can in turn activate cytoskeletal effectors (Strutt et al., 1997; Fanto et al., 2000; Bikkavilli et al., 2008) and transcriptional regulators. The latter are composed of the transcription factors cJun and cFos which act as dimers known as AP1 (Curran and Franza, 1988; Karin et al., 1997). We expressed a dominant-negative form of Jun kinase (Jnk^DN^) in a constitutive manner (Figure 5E) and used the temperature-driven conditional expression (48 h prior to repeated stimulations; Figures 5B,G). In both cases, we observed a significant decrease in ghost bouton formation. In the experiment in which we use constitutive expression, control preparations showed a mean of 6.82 ± 0.52 (Figure 5E) ghost boutons after stimulation, while the mean ghost bouton number of preparations with Jnk^DN^ constitutive expression was 2.47 ± 0.6 (Figure 5E; *p* < 0.0001). When we use the conditional expression system, the control synapses showed 8.97 ± 0.57 (Figures 5A,G) but only 5.63 ± 0.61 (Figures 5B,G; *p* = 0.0018) in preparations presenting Jnk^DN^ conditional expression. This shows that Jnk activity is required to fully achieve the morphological changes associated with ADSP.

We then logically asked whether the transcription factors cJun and cFos were involved in transducing the signal necessary for the ghost bouton formation after stimulation. To do so, we expressed dominant negative forms (Eresh et al., 1997) of these two constructs in a constitutive and conditional form (Figures 5C–G). We observed no difference between control preparations and preparations containing the dominant negative forms in unstimulated and stimulated procedures. When expressing the dominant negative constructs we noticed smaller synapses, a phenotype documented before (Ballard et al., 2014) that validates our expression system. This result suggests that the transcriptional control exercised by AP1 is not necessary for the morphological changes associated with activity-dependent synaptic plasticity. We favor the hypothesis that Jnk and the Rho GTPases act on cytoskeletal regulators to achieve these morphological changes.

## Discussion

Structural synaptic plasticity is a fundamental feature of the nervous system, in which Wg/Wnt signaling plays an essential role. Reduction of Wg and its receptor Fz2 impaired the formation of activity-dependent modifications of synaptic structure (Ataman et al., 2008; Alicea et al., 2017). Overexpression of GSK3β/Sgg, an important canonical pathway member, prevents ADSP at the Drosophila NMJ (Ataman et al., 2008). Upon stimulation, Wg protein release increases at the synapse (Ataman et al., 2008), which suggests an increased activation of Wg signaling pathways. Repeated simulation of the synapse also promotes an increase of CaMKII, and reducing synaptic CaMKII impairs the activity-dependent formation of *de novo* boutons (Nesler et al., 2016). This suggests some role of the Wnt/Ca^2+^ non-canonical pathway on activity-dependent synaptic plasticity.

Despite the existing interest in the Wnt/Wg PCP pathway, its role regarding processes of synaptic plasticity has not been extensively investigated. The Wg signal for polarity has been described extensively in several cellular environments (Maung and Jenny, 2011). In the developing eye, alteration of the PCP pathway provokes defects in the anterior–posterior and dorsal-ventral axes in the arrangement of ommatidia (Strutt et al., 1997; Boutros et al., 1998; Fanto et al., 2000; Adler, 2002; Strutt et al., 2013). In the Drosophila wing, the orientation of cellular hairs in the proximal-distal axis is impaired by defects of the PCP pathway (Strutt et al., 1997; Adler, 2002; Wu et al., 2013; Fagan et al., 2014). Neurons are one of the most morphologically polarized cell types, and mutations of Wnt/PCP pathway members like Dsh, Prickle, Strabismus, and Fz provoke axonal branching defects in mushroom bodies (MB) of *Drosophila* adult brain (Ng, 2012). Studies showed that Rac GTPase inactivation in mushroom bodies results in axonal branching defects (Ng et al., 2002). Wnt5, Fz, and Dsh act in concert with the small GTPase Rac1 to activate the actin assembly functions of dDAAM (dsh-associated activator of morphogenesis) necessary for the correct targeting of MB axons (Gombos et al., 2015). In addition, dDAAM regulates microtubule stability and synaptic growth at the *Drosophila* NMJ (Migh et al., 2018) while expression of Rac^DN^ (Park et al., 2022) and Rac^CA^ (Ball et al., 2010) promote synaptic overgrowth at the NMJ. Here, we have provided evidence that key molecules involved in the PCP pathway can affect the morphological modifications consequent to ADSP and are therefore necessary to achieve optimal activity-dependent synaptic plasticity. It is important to note that we never observed a complete ghost bouton elimination after affecting the PCP pathway. This makes sense since the other Wg pathways — the canonical pathway (Ataman et al., 2008; Alicea et al., 2017) and the calcium pathway (Freeman et al., 2011; Nesler et al., 2016)— are also necessary to achieve full ADSP. The contribution of the Wg PCP pathway to the formation of *de novo* synaptic structures after stimulation illustrates the redundancy and robustness of the Wg/Wnt signal in directing ADSP.

Our work revealed PCP pathway molecules exercising both activation and repression of ADSP. Indeed, the loss of function of *dsh*, either using RNAi or the PCP-specific allele *dsh^1^*, showed compromised plasticity. The same was true for the loss of function of *jnk*. This suggests that the role of these molecules is to promote activity. Activation and repression of ghost bouton formation was also achieved by modifying Rho GTPase activity. Expression of the constitutively active forms of the three GTPases we tested showed phenotypes, suggesting that these molecules can regulate ADSP. While Cdc42^CA^ and Rho1^CA^ expressing synapses show reduced plasticity, Rac1^CA^ expression elicited an over plastic response. This antagonistic relationship between Rho1 and Rac1 has been documented previously. For example, Rac1 activation promotes neurite formation and outgrowth, but Rho1 activation leads to neurite retraction and suppresses neurite outgrowth (Luo, 2000; Lorenzetto et al., 2013). Similarly, proper dendritic spine morphogenesis requires balanced Rho GTPase activity regulation (Penzes and Cahill, 2012). Interestingly, Rac1 induces the formation and maintenance of dendritic spines, while Rho1 decreases spine formation by promoting spine pruning or retraction (Nakayama et al., 2000; Tashiro et al., 2000; Newey et al., 2005; Bolognin et al., 2014). Like for neurite and spine formation but also cell migration (Ridley, 2015; Xu et al., 2019), our work has the potential to illustrate the importance of the relative balance of the GTPases’ spatiotemporal activity in regulating cytoskeletal dynamics.

Indeed, our work provides an insight into the complexity of the Rho GTPases at the NMJ. It is intriguing that the expression of the dominant negative or constitutively active forms of *cdc42* do not provide a phenotype when expressed since early embryogenesis. In contrast, both constructs’ expressions provoke a phenotype when expressed for 2 days before eliciting ADSP. This is reminiscent of the results obtained with Wg overexpression, where only a transient expression could influence synaptic proteins’ expression (Alicea et al., 2017). This perhaps illustrates that for Wg and its downstream GTPases, a briefer change of expression or activity likely better mimics the timescale relevant to ADSP than a constitutive expression. This result could be due to compensatory mechanisms between different GTPases. Indeed, the functional redundancy between Cdc42 and Rac1 is established. For example, Rac1 and Cdc42 both promote dendritic spine formation (Nakayama and Luo, 2000; Bolognin et al., 2014), cell extension (Nakayama et al., 2000; Swetman et al., 2002), and actin polymerization (Nobes and Hall, 1995; Lamarche et al., 1996). In addition, Cdc42 and Rac1 can be activated by the same molecular cascade (Buchsbaum, 2007) and there is evidence suggesting that depletion of Cdc42 in Hela cells can be compensated for by other GTPases to restore functional actin dynamic (Bonfim-Melo et al., 2018). Therefore, although there is evidence that GTPases functions are required at different times (Vadodaria et al., 2013), Rac1 could substitute Cdc42. The observed Cdc42 phenotypes indicate a role in repressing ADSP. Indeed, the dominant negative form is over plastic, while the constitutively active form is under plastic. This shows that in our system Cdc42 represses ghost bouton formation. This is surprising since most studies show that Cdc42 and the Rho GTPases in general promote membrane protrusion (Sadok and Marshall, 2014). Nevertheless, at the NMJ, *cdc42* mutations provoke an overgrown synapse, suggesting that Cdc42 represses synaptic growth (Rodal et al., 2008) while expression of Rac^DN^ (Park et al., 2022), Rac^CA^ and Rac^OE^ (Ball et al., 2010) promotes synaptic growth. These studies might indicate a more complex interaction between the Rho GTPases.

While Rho1^CA^ animals showed repressed ADSP, Rho1^DN^ did not show any phenotype. It is important to note that we know that Rho1^DN^ expression can decrease Rho function since its constitutive expression creates lethality. We can therefore hypothesize that the absence of phenotype in the Rho1^DN^ animals might be due to compensation/redundancy from other GTPase activity. Alternatively, its function at the time at which we express its dominant negative form is perhaps not to repress ADSP. Indeed, Rho GTPases serve different functions at different times. For example, in hippocampal neurogenesis, Cdc42 promotes initial dendritic development and spine maturation, while Rac1 is essential for the late stages of dendritic growth and spine maturation (Vadodaria et al., 2013).

Finally, both the constitutive and dominant negative forms of Rac1 provoke over plasticity at the NMJ. Although surprising, this is not the first time that the same phenotype has been observed when expressing these two constructs. Expression of both constitutively active and dominant-negative forms of Rac1 causes axonal loss in Drosophila (Luo et al., 1994), and Rac1 can have a dual function regulating attractive and repulsive signals in axonal guidance (Fan et al., 2003). Taken together, these results give an image of an intricate and complex local role of the Rho GTPases in regulating ADSP induced at the synapse upon high neuronal activity.

We have shown that the transcriptional regulator AP1 is unlikely to be involved in ADSP. We favor the hypothesis that regulation of the cytoskeleton by the PCP pathway is involved in regulating this plasticity. The reorganization of the synaptic cytoskeleton has been extensively studied in spines and is linked to processes of activity-dependent plasticity and learning and memory (Repetto et al., 2014; Macgillavry et al., 2016; Cornelius et al., 2021). This process involves the reorganization of filamentous actin, actin regulators, and microtubules (Ka and Kim, 2016; Schätzle et al., 2018; Cornelius et al., 2021). In fact, we have previously isolated one such regulator, the actin regulator Cortactin (Weed et al., 2000; Weaver et al., 2001) and showed that it is a regulator of ADSP, and that its protein is upregulated under the control of Wg signaling (Alicea et al., 2017). Determining whether the PCP pathway is involved in this regulation will be a challenge for the future.

## Data availability statement

The raw data supporting the conclusions of this article will be made available by the authors, without undue reservation.

## Ethics statement

Ethical approval was not required for the study involving animals in accordance with the local legislation and institutional requirements because our laboratory follows the guidelines required by the NIH and NSF granting agencies and uses ethical practices when handling our model system animal, the fruit fly *Drosophila melanogaster*. Our lab follows a Standard Operating Procedure (SOP) manual that was validated by the University. This document refers to and/or dictates the rules and lab conduct regarding safety and animals handling. It describes how to handle Drosophila properly and ethically.

## Author contributions

CD-C: Conceptualization, Formal Analysis, Investigation, Visualization, Writing – original draft, Writing – review & editing. MV: Investigation, Writing – review & editing. BM: Conceptualization, Funding acquisition, Supervision, Visualization, Writing – original draft, Writing – review & editing.

## References

[ref1] Adler P. N. (2002). Planar signaling and morphogenesis in *Drosophila*. Dev. Cell 2, 525–535. doi: 10.1016/s1534-5807(02)00176-4 12015961

[ref2] Alicea D. Perez M. Maldonado C. Dominicci-Cotto C. Marie B. (2017). Cortactin is a regulator of activity-dependent synaptic plasticity controlled by Wingless. J. Neurosci. 37, 2203–2215. doi: 10.1523/JNEUROSCI.1375-16.2017, PMID: 28123080 PMC5338761

[ref3] Ataman B. Ashley J. Gorczyca M. Ramachandran P. Fouquet W. Sigrist S. J.. (2008). Rapid activity-dependent modifications in synaptic structure and function require bidirectional Wnt signaling. Neuron 57, 705–718. doi: 10.1016/j.neuron.2008.01.026, PMID: 18341991 PMC2435264

[ref4] Auer M. Hausott B. Klimaschewski L. (2011). Rho GTPases as regulators of morphological neuroplasticity. Ann. Anat. 193, 259–266. doi: 10.1016/j.aanat.2011.02.015, PMID: 21459565 PMC3143277

[ref5] Axelrod J. D. Miller J. R. Shulman J. M. Moon R. T. Perrimon N. (1998). Differential recruitment of dishevelled provides signaling specificity in the planar cell polarity and Wingless signaling pathways. Genes Dev. 12, 2610–2622. doi: 10.1101/gad.12.16.2610, PMID: 9716412 PMC317102

[ref6] Bai Y. Suzuki T. (2020). Activity-dependent synaptic plasticity in *Drosophila melanogaster*. Front. Physiol. 11:161. doi: 10.3389/fphys.2020.00161, PMID: 32158405 PMC7052306

[ref8] Ballard S. L. Miller D. L. Ganetzky B. (2014). Retrograde neurotrophin signaling through Tollo regulates synaptic growth in *Drosophila*. J. Cell Biol. 204, 1157–1172. doi: 10.1083/JCB.201308115, PMID: 24662564 PMC3971753

[ref7] Ball R. W. Warren-Paquin M. Tsurudome K. Liao E. H. Elazzouzi F. Cavanagh C.. (2010). Retrograde BMP signaling controls synaptic growth at the nmj by regulating trio expression in motor neurons. Neuron 66, 536–549. doi: 10.1016/j.neuron.2010.04.011, PMID: 20510858

[ref9] Barrett K. Leptin M. Settleman J. (1997). The rho GTPase and a putative RhoGEF mediate a signaling pathway for the cell shape changes in *Drosophila* gastrulation. Cell 91, 905–915. doi: 10.1016/S0092-8674(00)80482-1, PMID: 9428514

[ref11] Bikkavilli R. K. Feigin M. E. Malbon C. C. (2008). Gαo mediates WNT-JNK signaling through dishevelled 1 and 3, RhoA family members, and MEKK 1 and 4 in mammalian cells. J. Cell Sci. 121, 234–245. doi: 10.1242/jcs.021964 18187455

[ref12] Billuart P. Winter C. G. Maresh A. Zhao X. Luo L. (2001). Regulating axon branch stability: the role of p190 RhoGAP in repressing a retraction signaling pathway. Cell 107, 195–207. doi: 10.1016/S0092-8674(01)00522-0 11672527

[ref13] Bolognin S. Lorenzetto E. Diana G. Buffelli M. (2014). The potential role of rho GTPases in Alzheimer’s disease pathogenesis. Mol. Neurobiol. 50, 406–422. doi: 10.1007/s12035-014-8637-5 24452387

[ref9001] Bonfim-Melo A. Ferreira É. R. Mortara R. A. (2018). Rac1/WAVE2 and Cdc42/N-WASP participation in actin-dependent host cell invasion by extracellular amastigotes of Trypanosoma cruzi. Front. microbiol. 9:360, 1–14. doi: 10.3389/fmicb.2018.00360 29541069 PMC5835522

[ref14] Boutros M. Paricio N. Strutt D. I. Mlodzik M. (1998). Dishevelled activates JNK and discriminates between JNK pathways in planar polarity and Wingless signaling. Cell 94, 109–118. doi: 10.1016/S0092-8674(00)81226-X, PMID: 9674432

[ref15] Brand A. H. Perrimon N. (1993). Targeted gene expression as a means of altering cell fates and generating dominant phenotypes. Development 118, 401–415. doi: 10.1242/dev.118.2.401, PMID: 8223268

[ref16] Brouns M. R. Matheson S. F. Settleman J. (2001). p190 RhoGAP is the principal Src substrate in brain and regulates axon outgrowth, guidance and fasciculation. Nat. Cell Biol. 3, 361–367. doi: 10.1038/35070042, PMID: 11283609

[ref17] Buchsbaum R. J. (2007). Rho activation at a glance. J. Cell Sci. 120, 1149–1152. doi: 10.1242/jcs.03428 17376960

[ref18] Budnik V. Koh Y. H. Guan B. Hartmann B. Hough C. Woods D.. (1996). Regulation of synapse structure and function by the Drosophila tumor suppressor gene dlg. Neuron 17, 627–640. doi: 10.1016/S0896-6273(00)80196-8, PMID: 8893021 PMC4661176

[ref19] Budnik V. Salinas P. C. (2011). Wnt signaling during synaptic development and plasticity. Curr. Opin. Neurobiol. 21, 151–159. doi: 10.1016/j.conb.2010.12.002, PMID: 21239163 PMC3499977

[ref20] Castillo P. E. (2012). Presynaptic LTP and LTD of excitatory and inhibitory synapses. Cold Spring Harb. Perspect. Biol. 4, 1–23. doi: 10.1101/cshperspect.a005728, PMID: 22147943 PMC3281573

[ref21] Chen J. Park C. S. Tang S.-J. (2006). Activity-dependent synaptic Wnt release regulates hippocampal long term potentiation. J. Biol. Chem. 281, 11910–11916. doi: 10.1074/jbc.M511920200, PMID: 16501258

[ref22] Ciani L. Boyle K. A. Dickins E. Sahores M. Anane D. Lopes D. M.. (2011). Wnt7a signaling promotes dendritic spine growth and synaptic strength through ca 2+/calmodulin-dependent protein kinase II. Proc. Natl. Acad. Sci. U. S. A. 108, 10732–10737. doi: 10.1073/pnas.1018132108, PMID: 21670302 PMC3127879

[ref23] Cornelius J. Rottner K. Korte M. Michaelsen-preusse K. (2021). Cortactin contributes to activity-dependent modulation of spine actin dynamics and spatial memory formation. Cells 10, 1–13. doi: 10.3390/cells10071835, PMID: 34360003 PMC8303107

[ref24] Coso O. A. Chiariello M. Yu J. C. Teramoto H. Crespo P. Xu N.. (1995). The small GTP-binding proteins Rac1 and Cdc42regulate the activity of the JNK/SAPK signaling pathway. Cell 81, 1137–1146. doi: 10.1016/S0092-8674(05)80018-2, PMID: 7600581

[ref25] Curran T. Franza B. R. (1988). Fos and Jun: the AP-1 connection. Cell 55, 395–397. doi: 10.1016/0092-8674(88)90024-4 3141060

[ref26] Dickson B. J. (2001). Rho GTPases in growth cone guidance. Curr. Opin. Neurobiol. 11, 103–110. doi: 10.1016/s0959-4388(00)00180-x 11179879

[ref27] Eresh S. Riese J. Jackson D. B. Bohmann D. Bienz M. (1997). A CREB-binding site as a target for decapentaplegic signalling during Drosophila endoderm induction. EMBO J. 16, 2014–2022. doi: 10.1093/emboj/16.8.2014, PMID: 9155027 PMC1169804

[ref28] Etter P. D. Narayanan R. Navratilova Z. Patel C. Bohmann D. Jasper H.. (2005). Synaptic and genomic responses to JNK and AP-1 signaling in Drosophila neurons. BMC Neurosci. 6:39. doi: 10.1186/1471-2202-6-39, PMID: 15932641 PMC1175850

[ref29] Fagan J. K. Dollar G. Lu Q. Barnett A. Jorge J. P. Schlosser A.. (2014). Combover/CG10732, a novel PCP effector for Drosophila wing hair formation. PLoS One 9:e107311. doi: 10.1371/JOURNAL.PONE.0107311, PMID: 25207969 PMC4160248

[ref31] Fanto M. Weber U. Strutt D. I. Mlodzik M. (2000). Nuclear signaling by Rac and rho GTPases is required in the establishment of epithelial planar polarity in the Drosophila eye. Curr. Biol. 10, 979–9S1. doi: 10.1016/S0960-9822(00)00645-X, PMID: 10985385

[ref30] Fan X. Labrador J. P. Hing H. Bashaw G. J. (2003). Slit stimulation recruits dock and Pak to the roundabout receptor and increases Rac activity to regulate axon repulsion at the CNS midline. Neuron 40, 113–127. doi: 10.1016/S0896-6273(03)00591-9, PMID: 14527437

[ref32] Ferrari M. E. Bernis M. E. McLeod F. Podpolny M. Coullery R. P. Casadei I. M.. (2018). Wnt7b signalling through Frizzled-7 receptor promotes dendrite development by coactivating CaMKII and JNK. J. Cell Sci. 131, 1–14. doi: 10.1242/jcs.216101, PMID: 29898920

[ref33] Freeman A. Franciscovich A. Bowers M. Sandstrom D. J. Sanyal S. (2011). NFAT regulates pre-synaptic development and activity-dependent plasticity in *Drosophila*. Mol. Cell. Neurosci. 46, 535–547. doi: 10.1016/j.mcn.2010.12.010, PMID: 21185939 PMC3030698

[ref34] Fuentes-Medel Y. Logan M. A. Ashley J. Ataman B. Budnik V. Freeman M. R. (2009). Glia and muscle sculpt neuromuscular arbors by engulfing destabilized synaptic boutons and shed presynaptic debris. PLoS Biol. 7:e1000184. doi: 10.1371/journal.pbio.1000184, PMID: 19707574 PMC2724735

[ref35] Gao C. Chen Y. G. (2010). Dishevelled: the hub of Wnt signaling. Cell. Signal. 22, 717–727. doi: 10.1016/j.cellsig.2009.11.021 20006983

[ref36] Gombos R. Migh E. Antal O. Mukherjee A. Jenny A. Mihaly J. (2015). The Formin DAAM functions as molecular effector of the planar cell polarity pathway during axonal development in Drosophila. J. Neurosci. 35, 10154–10167. doi: 10.1523/JNEUROSCI.3708-14.2015, PMID: 26180192 PMC4502256

[ref37] Hariharan I. K. Hu K. Q. Asha H. Quintanilla A. Ezzell R. M. Settleman J. (1995). Characterization of rho GTPase family homologues in *Drosophila melanogaster*: overexpressing Rho1 in retinal cells causes a late developmental defect. EMBO J. 14, 292–302. doi: 10.1002/j.1460-2075.1995.tb07003.x, PMID: 7835340 PMC398083

[ref38] Harris K. P. Littleton J. T. (2015). Transmission, development, and plasticity of synapses. Genetics 201, 345–375. doi: 10.1534/GENETICS.115.176529, PMID: 26447126 PMC4596655

[ref40] Holtmaat A. Svoboda K. (2009). Experience-dependent structural synaptic plasticity in the mammalian brain. Nat. Rev. Neurosci. 10, 647–658. doi: 10.1038/nrn2699 19693029

[ref39] Ho V. M. Lee J. A. Martin K. C. (2011). The cell biology of synaptic plasticity. Science 334, 623–628. doi: 10.1126/science.1209236, PMID: 22053042 PMC3286636

[ref41] Jaffe A. B. Hall A. (2005). Rho GTPases: biochemistry and biology. Ann. Rev. Cell Develop. Biol. 21, 247–269. doi: 10.1146/annurev.cellbio.21.020604.150721 16212495

[ref42] Jan L. Y. Jan Y. N. (1982). Antibodies to horseradish peroxidase as specific neuronal markers in Drosophila and in grasshopper embryos. Proc. Natl. Acad. Sci. U. S. A. 79, 2700–2704. doi: 10.1073/pnas.79.8.2700, PMID: 6806816 PMC346269

[ref43] Jenny A. (2010). Planar cell polarity signaling in the *Drosophila* eye. Curr. Topics Develop. Biol 93, 189–227. doi: 10.1016/B978-0-12-385044-7.00007-2, PMID: 20959167 PMC5310647

[ref44] Ka M. Kim W. Y. (2016). Microtubule-actin crosslinking factor 1 is required for dendritic Arborization and axon outgrowth in the developing brain. Mol. Neurobiol. 53, 6018–6032. doi: 10.1007/s12035-015-9508-4, PMID: 26526844 PMC4853297

[ref45] Karin M. Liu Z. G. Zandi E. (1997). AP-1 function and regulation. Curr. Opin. Cell Biol. 9, 240–246. doi: 10.1016/S0955-0674(97)80068-3 9069263

[ref46] Kaufmann N. Wills Z. P. Van Vactor D. (1998). Drosophila Rac1 controls motor axon guidance. Development 125, 453–461. doi: 10.1242/dev.125.3.453, PMID: 9425140

[ref47] Klingensmith J. Nusse R. Perrimon N. (1994). The Drosophila segment polarity gene dishevelled encodes a novel protein required for response to the Wingless signal. Genes Dev. 8, 118–130. doi: 10.1101/gad.8.1.118, PMID: 8288125

[ref48] Koles K. Budnik V. (2012). Wnt signaling in neuromuscular junction development. Cold Spring Harb. Perspect. Biol. 4, 1–22. doi: 10.1101/cshperspect.a008045, PMID: 22510459 PMC3367558

[ref49] Lahey T. Gorczyca M. Jia X. X. Budnik V. (1994). The Drosophila tumor suppressor gene dlg is required for normal synaptic Bouton structure. Neuron 13, 823–835. doi: 10.1016/0896-6273(94)90249-6, PMID: 7946331 PMC4661177

[ref50] Lamarche N. Tapon N. Stowers L. Burbelo P. D. Aspenström P. Bridges T.. (1996). Rac and Cdc42 induce actin polymerization and G1 cell cycle progression independently of p65PAK and the JNK/SAPK MAP kinase cascade. Cell 87, 519–529. doi: 10.1016/S0092-8674(00)81371-9, PMID: 8898204

[ref51] Lee J. Y. Geng J. Lee J. Wang A. R. Chang K. T. (2017). Activity-induced synaptic structural modifications by an activator of integrin signaling at the Drosophila neuromuscular junction. J. Neurosci. 37, 3246–3263. doi: 10.1523/JNEUROSCI.3128-16.2017, PMID: 28219985 PMC5373117

[ref52] Li X. Wang Y. Wang H. Liu T. Guo J. Yi W.. (2016). Epithelia-derived Wingless regulates dendrite directional growth of drosophila ddaE neuron through the Fz-Fmi-Dsh-Rac1 pathway. Mol. Brain 9, 1–15. doi: 10.1186/S13041-016-0228-0, PMID: 27129721 PMC4850637

[ref53] Lorenzetto E. Ettorre M. Pontelli V. Bolomini-Vittori M. Bolognin S. Zorzan S.. (2013). Rac1 selective activation improves retina ganglion cell survival and regeneration. PLoS One 8:e64350. doi: 10.1371/journal.pone.0064350, PMID: 23734197 PMC3667179

[ref54] Luo L. (2000). Rho GTPases in neuronal morphogenesis. Nat. Rev. Neurosci. 1, 173–180. doi: 10.1038/35044547 11257905

[ref55] Luo L. (2002). Actin cytoskeleton regulation in neuronal morphogenesis and structural plasticity. Annu. Rev. Cell Dev. Biol. 18, 601–635. doi: 10.1146/annurev.cellbio.18.031802.150501 12142283

[ref56] Luo L. Joyce Liao Y. Jan L. Y. Jan Y. N. (1994). Distinct morphogenetic functions of similar small GTPases: Drosophila Drac1 is involved in axonal outgrowth and myoblast fusion. Genes Dev. 8, 1787–1802. doi: 10.1101/gad.8.15.1787, PMID: 7958857

[ref57] Lüscher C. Malenka R. C. (2012). NMDA receptor-dependent long-term potentiation and long-term depression (LTP/LTD). Cold Spring Harb. Perspect. Biol. 4, 1–15. doi: 10.1101/cshperspect.a005710, PMID: 22510460 PMC3367554

[ref58] Macgillavry H. D. Kerr J. M. Kassner J. Frost N. A. Blanpied T. A. (2016). Shank-cortactin interactions control actin dynamics to maintain flexibility of neuronal spines and synapses. Eur. J. Neurosci. 43, 179–193. doi: 10.1111/ejn.13129, PMID: 26547831 PMC5007541

[ref59] Maldonado-Díaz C. Vazquez M. Marie B. (2021). A comparison of three different methods of eliciting rapid activity-dependent synaptic plasticity at the Drosophila NMJ. PLoS One 16:e0260553. doi: 10.1371/journal.pone.0260553, PMID: 34847197 PMC8631638

[ref60] Maung S. M. T. W. Jenny A. (2011). Planar cell polarity in *Drosophila*. Organogenesis 7, 165–179. doi: 10.4161/ORG.7.3.18143, PMID: 21983142 PMC3243030

[ref62] McGuire S. E. Mao Z. Davis R. L. (2004). Spatiotemporal gene expression targeting with the TARGET and gene-switch systems in *Drosophila*. Sci. STKE 2004, 1–10. doi: 10.1126/stke.2202004pl6 14970377

[ref63] McLeod F. Bossio A. Marzo A. Ciani L. Sibilla S. Hannan S.. (2018). Wnt signaling mediates LTP-dependent spine plasticity and AMPAR localization through Frizzled-7 receptors. Cell Rep. 23, 1060–1071. doi: 10.1016/j.celrep.2018.03.119, PMID: 29694885 PMC5946458

[ref64] Migh E. Götz T. Földi I. Szikora S. Gombos R. Darula Z.. (2018). Microtubule organization in presynaptic boutons relies on the formin DAAM. Development (Cambridge) 145, 1–13. doi: 10.1242/dev.158519, PMID: 29487108

[ref65] Moriguchi T. Kawachi K. Kamakura S. Masuyama N. Yamanaka H. Matsumoto K.. (1999). Distinct domains of mouse dishevelled are responsible for the c-Jun N-terminal kinase/stress-activated protein kinase activation and the Axis formation in vertebrates. J. Biol. Chem. 274, 30957–30962. doi: 10.1074/JBC.274.43.30957, PMID: 10521491

[ref67] Nakayama A. Y. Harms M. B. Luo L. (2000). Small GTPases Rac and rho in the maintenance of dendritic spines and branches in hippocampal pyramidal neurons. J. Neurosci. 20, 5329–5338. doi: 10.1523/jneurosci.20-14-05329.2000, PMID: 10884317 PMC6772334

[ref68] Nakayama A. Y. Luo L. (2000). Intracellular signalling pathways that regulate dendritic spine morphogenesis. Hippocampus 10, 582–586. doi: 10.1002/1098-1063(2000)10:5<582::AID-HIPO8>3.0.CO;2-K 11075828

[ref69] Nesler K. R. Sand R. I. Symmes B. A. Pradhan S. J. Boin N. G. Laun A. E.. (2013). The miRNA pathway controls rapid changes in activity-dependent synaptic structure at the *Drosophila melanogaster* neuromuscular junction. PLoS One 8:e68385. doi: 10.1371/journal.pone.0068385, PMID: 23844193 PMC3699548

[ref70] Nesler K. R. Starke E. L. Boin N. G. Ritz M. Barbee S. A. (2016). Presynaptic CamKII regulates activity-dependent axon terminal growth. Mol. Cell. Neurosci. 76, 33–41. doi: 10.1016/j.mcn.2016.08.007, PMID: 27567686 PMC5056856

[ref9002] Newey S. E. Velamoor V. Govek E. E. Van Aelst L. (2005). Rho GTPases, dendritic structure, and mental retardation. J. Neurobiol. 64, 58–74. doi: 10.1002/neu.20153 15884002

[ref71] Ng J. (2012). Wnt/PCP proteins regulate stereotyped axon branch extension in Drosophila. Development 139, 165–177. doi: 10.1242/dev.068668, PMID: 22147954 PMC3231775

[ref72] Ng J. Nardine T. Harms M. Tzu J. Goldstein A. Sun Y.. (2002). Rac GTPases control axon growth, guidance and branching. Nature 416, 442–447. doi: 10.1038/416442a, PMID: 11919635

[ref73] Nobes C. D. Hall A. (1995). Rho, Rac, and Cdc42 GTPases regulate the assembly of multimolecular focal complexes associated with actin stress fibers, lamellipodia, and filopodia. Cell 81, 53–62. doi: 10.1016/0092-8674(95)90370-4, PMID: 7536630

[ref74] Ohnami S. Endo M. Hirai S. Uesaka N. Hatanaka Y. Yamashita T.. (2008). Role of RhoA in activity-dependent cortical axon branching. J. Neurosci. 28, 9117–9121. doi: 10.1523/JNEUROSCI.1731-08.2008, PMID: 18784292 PMC6670927

[ref75] Olson M. F. Ashworth A. Hall A. (1995). An essential role for rho, Rac, and Cdc42 GTPases in cell cycle progression through G1. Science 269, 1270–1272. doi: 10.1126/science.7652575, PMID: 7652575

[ref76] Park H. G. Kim Y. D. Cho E. Lu T. Y. Yao C. K. Lee J.. (2022). Vav independently regulates synaptic growth and plasticity through distinct actin-based processes. J. Cell Biol. 221, 1–21. doi: 10.1083/jcb.202203048, PMID: 35976098 PMC9388202

[ref77] Penzes P. Cahill M. E. (2012). Deconstructing signal transduction pathways that regulate the actin cytoskeleton in dendritic spines. Cytoskeleton 69, 426–441. doi: 10.1002/cm.21015, PMID: 22307832 PMC3576145

[ref78] Pereda A. E. (2014). Electrical synapses and their functional interactions with chemical synapses. Nat. Rev. Neurosci. 15, 250–263. doi: 10.1038/nrn3708, PMID: 24619342 PMC4091911

[ref79] Perrimon N. Mahowald A. P. (1987). Multiple functions of segment polarity genes in Drosophila. Dev. Biol. 119, 587–600. doi: 10.1016/0012-1606(87)90061-3 3803719

[ref80] Piccin A. Salameh A. Benna C. Sandrelli F. Mazzotta G. Zordan M.. (2001). Efficient and heritable functional knock-out of an adult phenotype in Drosophila using a GAL4-driven hairpin RNA incorporating a heterologous spacer. Nucleic Acids Res. 29:55e. doi: 10.1093/NAR/29.12.E55, PMID: 11410678 PMC55754

[ref81] Piccioli Z. D. Littleton J. T. (2014). Retrograde BMP signaling modulates rapid activity-dependent synaptic growth via presynaptic lim kinase regulation of Cofilin. J. Neurosci. 34, 4371–4381. doi: 10.1523/JNEUROSCI.4943-13.2014, PMID: 24647957 PMC3960475

[ref82] Repetto D. Camera P. Melani R. Morello N. Russo I. Calcagno E.. (2014). p140Cap regulates memory and synaptic plasticity through Src-mediated and citron-N-mediated actin reorganization. J. Neurosci. 34, 1542–1553. doi: 10.1523/JNEUROSCI.2341-13.2014, PMID: 24453341 PMC6705312

[ref83] Ridley A. J. (2015). Rho GTPase signalling in cell migration. Curr. Opinion Cell Biol. 36, 103–112. doi: 10.1016/j.ceb.2015.08.005, PMID: 26363959 PMC4728192

[ref84] Robinow S. White K. (1991). Characterization and spatial distribution of the ELAV protein during *Drosophila melanogaster* development. J. Neurobiol. 22, 443–461. doi: 10.1002/neu.480220503, PMID: 1716300

[ref85] Rodal A. A. Motola-Barnes R. N. Littleton J. T. (2008). Nervous wreck and Cdc42 cooperate to regulate endocytic actin assembly during synaptic growth. J. Neurosci. 28, 8316–8325. doi: 10.1523/JNEUROSCI.2304-08.2008, PMID: 18701694 PMC2546611

[ref86] Rosso S. B. Inestrosa N. C. Rosso S. B. (2013). WNT signaling in neuronal maturation and synaptogenesis. Front. Cell. Neurosci. 7:103. doi: 10.3389/fncel.2013.00103, PMID: 23847469 PMC3701138

[ref87] Rosso S. B. Sussman D. Wynshaw-Boris A. Salinas P. C. (2005). Wnt signaling through Dishevelled, Rac and JNK regulates dendritic development. Nat. Neurosci. 8, 34–42. doi: 10.1038/nn1374, PMID: 15608632

[ref89] Sadok A. Marshall C. J. (2014). Rho gtpases masters of cell migration. Small GTPases 5:e983878. doi: 10.4161/sgtp.29710, PMID: 24978113 PMC4107589

[ref90] Sanyal S. Sandstrom D. J. Hoeffer C. A. Ramaswami M. (2002). Ap-1 functions upstream of CREB to control synaptic plasticity in drosophila. Nature 416, 870–874. doi: 10.1038/416870a, PMID: 11976688

[ref91] Schätzle P. Esteves da Silva M. Tas R. P. Katrukha E. A. Hu H. Y. Wierenga C. J.. (2018). Activity-dependent actin remodeling at the base of dendritic spines promotes microtubule entry. Curr. Biol. 28, 2081–2093.e6. doi: 10.1016/j.cub.2018.05.004, PMID: 29910073

[ref93] Schlessinger K. McManus E. J. Hall A. (2007). Cdc42 and noncanonical Wnt signal transduction pathways cooperate to promote cell polarity. J. Cell Biol. 178, 355–361. doi: 10.1083/jcb.200701083, PMID: 17646398 PMC2064837

[ref94] Shepherd G. M. (ed.). (2004). “Introduction to synaptic circuits” in The synaptic Organization of the Brain. *5th ed*. (New York: Oxford University Press).

[ref96] Strutt D. I. Weber U. Mlodzik M. (1997). The role of RhoA in tissue polarity and frizzled signalling. Nature 387, 292–295. doi: 10.1038/387292a0, PMID: 9153394

[ref95] Strutt H. Searle E. Thomas-Macarthur V. Brookfield R. Strutt D. (2013). A Cul-3-BTB ubiquitylation pathway regulates junctional levels and asymmetry of core planar polarity proteins. Development 140, 1693–1702. doi: 10.1242/dev.089656, PMID: 23487316 PMC3621487

[ref97] Swetman C. A. Leverrier Y. Garg R. Gan C. H. V. Ridley A. J. Katz D. R.. (2002). Extension, retraction and contraction in the formation of a dendritic cell dendrite: distinct roles for rho GTPases. Eur. J. Immunol. 32, 2074–2083. doi: 10.1002/1521-4141(200207)32:7<2074::AID-IMMU2074>3.0.CO;2-S, PMID: 12115629

[ref98] Tabatadze N. Mcgonigal R. Neve R. L. Routtenberg A. (2014). Activity-dependent Wnt 7 dendritic targeting in hippocampal neurons: plasticity- and tagging-related retrograde signaling mechanism? Hippocampus 24, 455–465. doi: 10.1002/hipo.22239, PMID: 24375790

[ref9003] Tashiro A. Minden A. Yuste R. (2000). Regulation of dendritic spine morphology by the rho family of small GTPases: antagonistic roles of Rac and Rho. Cereb. Cortex. 10, 927–938. doi: 10.1093/cercor/10.10.927 11007543

[ref99] Vadodaria K. C. Brakebusch C. Suter U. Jessberger S. (2013). Stage-specific functions of the small rho GTPases Cdc42 and Rac1 for adult hippocampal neurogenesis. J. Neurosci. 33, 1179–1189. doi: 10.1523/JNEUROSCI.2103-12.2013, PMID: 23325254 PMC6704849

[ref100] Vasin A. Sabeva N. Torres C. Phan S. Bushong E. A. Ellisman M. H.. (2019). Two pathways for the activity-dependent growth and differentiation of synaptic boutons in drosophila. ENeuro 6, ENEURO.0060–ENEU19.2019. doi: 10.1523/ENEURO.0060-19.2019, PMID: 31387877 PMC6709223

[ref101] Vasin A. Zueva L. Torrez C. Volfson D. Troy Littleton J. Bykhovskaia M. (2014). Synapsin regulates activity-dependent outgrowth of synaptic boutons at the Drosophila neuromuscular junction. J. Neurosci. 34, 10554–10563. doi: 10.1523/JNEUROSCI.5074-13.2014, PMID: 25100589 PMC4200108

[ref102] Wayman G. A. Impey S. Marks D. Saneyoshi T. Grant W. F. Derkach V.. (2006). Activity-dependent dendritic Arborization mediated by CaM-kinase I activation and enhanced CREB-dependent transcription of Wnt-2. Neuron 50, 897–909. doi: 10.1016/j.neuron.2006.05.008, PMID: 16772171

[ref103] Weaver A. M. Karginov A. V. Kinley A. W. Weed S. A. Li Y. Parsons J. T.. (2001). Cortactin promotes and stabilizes Arp2/3-induced actin filament network formation. Curr. Biol. 11, 370–374. doi: 10.1016/S0960-9822(01)00098-7, PMID: 11267876

[ref104] Weed S. A. Karginov A. V. Schafer D. A. Weaver A. M. Kinley A. W. Cooper J. A.. (2000). Cortactin localization to sites of actin assembly in Lamellipodia requires interactions with F-actin and the Arp2/3 complex. J. Cell Biol. 151, 29–40. doi: 10.1083/JCB.151.1.29, PMID: 11018051 PMC2189811

[ref105] Wu G. Y. Deisseroth K. Tsien R. W. (2001). Spaced stimuli stabilize MAPK pathway activation and its effects on dendritic morphology. Nat. Neurosci. 4, 151–158. doi: 10.1038/83976, PMID: 11175875

[ref106] Wu J. Roman A. C. Carvajal-Gonzalez J. M. Mlodzik M. (2013). Wg and Wnt4 provide long-range directional input to planar cell polarity orientation in Drosophila. Nat. Cell Biol. 15, 1045–1055. doi: 10.1038/ncb2806, PMID: 23912125 PMC3762953

[ref107] Xu Z. Chen Y. Chen Y. (2019). Spatiotemporal regulation of rho GTPases in neuronal migration. Cells 8:568. doi: 10.3390/cells8060568, PMID: 31185627 PMC6627650

[ref109] Yanfeng W. A. Berhane H. Mola M. Singh J. Jenny A. Mlodzik M. (2011). Functional dissection of phosphorylation of disheveled in Drosophila. Dev. Biol. 360, 132–142. doi: 10.1016/j.ydbio.2011.09.017, PMID: 21963539 PMC3221411

[ref110] Yu X. Malenka R. C. (2003). β-Catenin is critical for dendritic morphogenesis. Nat. Neurosci. 6, 1169–1177. doi: 10.1038/nn1132, PMID: 14528308

